# Gastroprotective Effect of *Rubia cordifolia* Linn. on Aspirin Plus Pylorus-Ligated Ulcer

**DOI:** 10.1155/2011/541624

**Published:** 2011-02-24

**Authors:** R. S. Deoda, Dinesh Kumar, S. S. Bhujbal

**Affiliations:** ^1^Marathwada Mitra Mandal's College of Pharmacy, Thergaon, Kalewadi, Pune 17, India; ^2^Department of Pharmaceutical Sciences, University of Kashmir, Srinagar 190006, India; ^3^Padam Shree Dr. D. Y. Patil Institute of Pharmaceutical Sciences and Research, Pimpri, Pune 18, India

## Abstract

The present study was designed to investigate the effect of *Rubia cordifolia* (Rubiaceae) against experimentally induced gastric ulcer and compare activity with its fractions by employing aspirin plus pylorus-ligated ulcer screening model in Wistar rats. Total acidity, volume of gastric acid secretion, total acid output, and pepsin activity show significant reduction, when compared with the control group. The present study confirmed that chloroform fraction showed the significant activity at lower doses compared to parent extract. The mechanism can be attributed to decrease in gastric acid secretary activity along with strengthening of mucosal defensive mechanism by prostaglandin synthesis and antioxidant potential.

## 1. Introduction

*Rubia cordifolia* Linn. (Rubiaceae) was popularly known as “Indian madder”. It is an important ingredient of many ayurvedic preparations. Roots are traditionally used as anti-inflammatory, antiulcer, antidysenteric, and blood purifier, as well as various chemical constituents like anthraquinones, iridoid glycoside, bicyclic hexapeptides, and triterpenes have been isolated and identified from the plant [1]. Peptic ulcer is one of the major gastrointestinal disorders, which occur due to an imbalance between the offensive (gastric acid secretion) and defensive (gastric mucosal integrity) factors [2]. Consequently, reduction of gastric acid production as well as reinforcement of gastric mucosal production has been the major approaches for therapy of peptic ulcer disease. Prostaglandins are synthesized by the gastric mucosa and are known to inhibit the secretion of gastric acid and stimulate the secretion of mucus and bicarbonate. Also, there is evidence concerning the participation of reactive oxygen species in the etiology and pathophysiology of ulcer [3]. The objective of the present study was to investigate the gastroprotective and antioxidant activities of methanolic extract of roots of *R. cordifolia* and its chloroform and residual fractions by using aspirin plus pylorus ligation model.

## 2. Material and Methods

### 2.1. Plant Material and Extraction

Roots of *Rubia cordifolia* were collected from Bhimashankar Hills (Ghat regions of the sahyandri hills), Taluka Khed, District Pune, and Maharashtra, in the month of August. The plant was identified and authenticated from Regional Research Institute (AY) Kothrud, Pune, by Dr. (Mr.) Rajesh Dabur, research officer, as Specimen Voucher no. 645 was deposited at the herbarium of the RRI for future reference. 

It was shade dried, powdered, and extracted with methanol. The yield was 12.5%. It was then vacuum dried, and half of the methanolic extract was successively fractionate with chloroform (yield = 24.7%) using continuous soxhlet apparatus. Parent extract (ME), along with chloroform fraction (CF) and its residual fraction (RF), was used for the further experimental model.

### 2.2. Drugs and Chemicals

Alcian blue, disodium hydrogen phosphate, 5,5-dithio bis(2-nitro Benzoic acid), EDTA, magnesium chloride hexahydrate crystals, phenolphthalein, sodium acetate trihydrate, sodium bicarbonate, sucrose, tris buffer tris buffer (Research Lab, Mumbai), bovine serum albumin (Himedia Pvt. Ltd., Mumbai), copper sulphate (Qualigens Fine Chemicals, Mumbai), Folin-Ciocalteu's phenol reagent (Loba Chemicals, Mumbai), potassium dihydrogen orthophosphate (Pure Chemical. Laboratories, Pune), sodium carbonate anhydrous (Qualigens Fine Chemicals, Mumbai), thiobarbituric acid (Spectrochem Pvt. Ltd., Bombay) trichloroacetic acid (Universal Laboratories Pvt. Ltd., Mumbai), tyrosine (Central Drug House P. Ltd., Bombay) saline (Mareck Hariyala, Gujarat) ketamine hydrochloride injection (Themis Medicare Ltd., Uttarakhand), aspirin (USV Ltd., Mumbai), and ranitidine (GlaxoSmithKline Pharmaceuticals Ltd., Mumbai)

### 2.3. Animals

Wistar rats weighing between 150–225 g were used for the study. Animals were maintained under controlled conditions of temperature 26 ± 2°C, relative humidity 44%–56%, and photoschedule (12 hr light and 12 hr dark). Animals were provided with standard pellet diet (Amrut Feeds, Mumbai, India) and water *ad libitum*. Institutional Animal Ethics Committee approved the experimental protocol (198/99/CPCSEA). The pharmacological work was carried out as per norms of CPCSEA (Committee for the Purpose of Control and Supervision of Experiments on Animals).

### 2.4. Acute Toxicity Studies

Rats were kept overnight fasting prior to drug administration. A total of nine animals were divided in three groups of three animals in each, which received a single oral dose (2000 mg/kg, b.w.) of methanolic extract of roots of *Rubia cordifolia *(ME), its chloroform fraction (CF), and residual fraction (RF), respectively. After the administration of ME, CF, and RF, animals were observed individually and continuously for 30 min, 2 hr, and 24 hr after dosing, periodically (with special attention during the first 4 h) and daily thereafter for a period of 14 days. Once daily cage side observations included changes in skin and fur, eyes, and mucous membrane (nasal) and also respiratory rate, circulatory (heart rate and blood pressure), autonomic (salivation, lacrimation, perspiration, piloerection, urinary incontinence, and defecation), and central nervous system (ptosis, drowsiness, gait, tremors, and convulsion) changes. Mortality, if any, was determined over a period of 2 weeks [4].

### 2.5. Selection of Dose of the Extract

LD_50_ was done as per OECD guidelines for fixing the dose for biological evaluation. The LD_50_ of the extract as per OECD guidelines falls under class four values with no signs of acute toxicity at 2000 mg/kg. The biological evaluations of ME was carried out at doses of 100, 200, and 400 mg/kg body weight, and CF and RF are the fractions of ME, and it was given in the doses of 50, 100, and 200 mg/kg body weight, to compare the effect of fractions against parent extract in low doses.

### 2.6. Experimental Procedure

#### 2.6.1. Study of Antiulcer Activity Using Aspirin Plus Pylorus Ligation Method

Methanolic extract, its chloroform and residual fraction of *Rubia cordifolia*, aspirin, and standard antiulcer drug, ranitidine, were prepared in 1% sodium carboxy methyl cellulose (NaCMC) suspension as vehicle and administered orally once daily at a volume of 10 ml/kg body weight. The animals were divided into twelve groups, consisting of six each. Group I represented the normal control group, which received 10 ml/kg body weight of vehicle (NaCMC 1%, p.o.). Group II represented the ulcerated control group, which received aspirin alone (200 mg/kg, p.o.). Group III represented the standard group, which received ranitidine orally at the dose of 10 mg/kg body weight, for 7 days. Groups IV, V, and VI received ME orally at the doses of 100, 200, and 400 mg/kg body weight, respectively, Groups VII, VIII, and IX received CF orally at the doses of 50,100, and 200 mg/kg body weight, respectively, Groups X, XI, and XII received RF orally at the doses of 50,100, and 200 mg/kg body weight, respectively, for 7 days. From days 5 to 7, animals of all the groups received aspirin orally as an aqueous suspension at a dose of 200 mg/kg, 2 hr after the administration of respective drug treatment. Animals in all the groups were fasted for 18 hr after the respective assigned treatment and were anaesthetised with ketamine hydrochloride (50 mg/kg, i.p.). Four hours after pylorus ligation, the rats were sacrificed and the stomach was removed and observed for ulcers [5].

Ulcer score: the numbers of ulcers were counted using magnifying lenses. Each ulcer was then measured with a vernier calliper to assess the diameter. Ulcer index was determined by scoring method of Suzuki et al. (1976). The percent protection with each test drug dose was also calculated by the following formula [6]:

(1) $$\%\;\text{Protection}=\frac{\left(\text{UI}\;\text{control}-\text{UI}\;\text{treated}\right)\times 100}{\left(\text{UI}\;\text{control}\right)},$$

where UI stands for ulcer index.

### 2.7. Effects on Gastric Acid Secretion

The gastric content of each stomach obtained from the pylorus was centrifuged at 2500 rpm for 20 min at 4°C; the volume of the supernatant (mL) and the pH value were measured. The volume was expressed as ml per 100 g b.w. [7]. An aliquot of 1 mL gastric juice was used to determine acidity and was expressed as meq/L [8].The peptic activity was determined by using bovine serum albumin as a substrate and expressed in terms of the amount (*μ*moles) of liberated L-tyrosine [7]. The protein content was estimated with Folin's phenol reagent at 750 nm [9].

### 2.8. Gastric Wall Mucus (Barrier Mucus) Determination

The glandular segments from stomach was transferred immediately to 10 mL of 0.1%, w : v alcian blue solution. After immersion for 2 hr, excess dye was removed by two successive rinses with 10 mL of 0.25 M sucrose, first for 15 min and then for 45 min. Dye complexed with gastric wall mucus was extracted with10 mL of 0.5 M magnesium chloride by shaking intermittently for 1 min at 30 mins intervals for 2 h. The resulting blue solution was shaken vigorously with an equal volume of diethyl ether, and then the emulsion was centrifuged at 3000 rpm for 10 min, and the absorbance of the aqueous layer against blank standard MgCl_2_ solution was recorded at 580 nm. The quantity of alcian blue recovered from per gram of net glandular tissue was then calculated [10].

### 2.9. Study of Antioxidant Activity

The animals were then sacrificed, and the stomach was removed, processed, and homogenized in Tris buffer (10 mM, pH 7.4) at a concentration of 10% (w/v). The homogenates were centrifuged at 10,000 × g at 4°C for 20 min, using Remi C-24 high-speed cooling centrifuge. The clear supernatant was used for the assays of lipid peroxidation (MDA content), endogenous antioxidant enzymes superoxide dismutase (SOD) and catalase (CAT) and reduced glutathione (GSH) [3].

### 2.10. Statistical Analysis

Results of all above estimations have been expressed in terms of mean ± SEM. Test and standard groups were compared with control group. Statistical comparison was performed using analysis of variance (ANOVA) followed by Turkey's test.

## 3. Results

### 3.1. Study of Gastroprotective Effect on Aspirin plus Pylorus Ligation Method

The ulcerated control group showed the ulcer index 80.63 ± 3.88, and the maximum numbers of ulcers were of the ulcer score 5 and 10, and also a number of perforated ulcers (Score 25) were observed.

Methanolic extract of *R. Cordifolia *was found to produce a decrease in ulcer index in the of 400 mg/kg dose. But chloroform fraction (CF) found to produce significant (*P* < .001) decrease in the ulcer index in all three doses, and residual fraction (RF) showed nonsignificant changes in the ulcer index. The total acidity and total acid output were also significantly (*P* < .01) reduced in all three doses of CF as compared to pylorus-ligated control group. Control group showed significant (*P* < .001) decrease in mucin content compared to normal group. CF showed maximum significant (*P* < .001) increase in mucin content of stomach as compared to ME (*P* < .01), whereas RF showed significant change at higher dose only. ME and CF groups showed a significant (*P* < .001) decrease in the pepsin and protein content compared to control group. RF group shows nonsignificant results for protein content and significance in pepsin content at higher dose only (Table 1).

### 3.2. Study of In Vivo Antioxidant Activity

Aspirin plus pylorus-ligated control group was found to increase lipid peroxidation and decrease SOD, catalase, and reduced glutathione levels, thus leading to oxidative stress. ME and CF showed significant (*P* < .001) reduction in lipid peroxidation and increase in the catalase, SOD, and content of reduced glutathione.

Ranitidine (10 mg/kg) was found to produce significant (*P* < .001) reduction in ulcer index. It also reduces the total acidity, total acid output, pepsin content, and protein content, whereas mucin content increased as compared to the pylorus-ligated control group. It also showed significant (*P* < .001) increase in the SOD, catalase, and reduced glutathione, whereas no as such effect on lipid peroxidation (Table 2).

## 4. Discussion and Conclusions

The use of various herbal medicines for various disorders is now widely accepted. However, both in the clinical practice and diagnostic and therapeutic, these systems fail to find exacting correlates with those in modern medicines. The disease classification is essentially based on symptomatology and therapy designed with varied preoccupations. It becomes difficult, therefore, to define with certainty the pharmacological activity to be evaluated with respect to the traditionally prescribed use of an indigenous drug. Nevertheless, presumptions based on nature of traditional use and evaluation of specific beneficial activity of indigenous drugs have been found to be successful approach in medicinal plant research [8]. 

The *Rubia cordifolia* has been reported the presence of alkaloids, phytosterols, saponins, tannins, carbohydrates, phenolic compounds, anthraquinone glycosides, and triterpenoids [1]. The chloroform fraction mainly contains triterpenoids in major concentration [11]. Pentacyclic triterpenoids, in addition to their anti-inflammatory properties, are also known to promote mucus secretion. Thus, mucus secreting potential and subsequent wound healing effect of the extract of *Rubia cordifolia* may be linked to the presence of triterpenes. In addition, triterpenoids are also reported to be good antioxidant, such as *α*-and *β*-amirins, oleanolic acid, ursolic acid, and lupeol and glycirretinic acid [12]. Oleananes, rubicoumaric acid, rubifolic acid, and various other types of triterpenoids were isolated from chloroform fraction of methanolic extract of roots of *Rubia cordifolia* [11, 13].

Aspirin causes mucosal damage by interfering with prostaglandin synthesis. Disturbances in gastric secretion, damage to gastric mucosa, alteration in permeability, gastric mucus depletion, increase in the pepsin and protein content, and generation of free radical production are reported to be the pathogenic effects of aspirin plus pylorus-ligated ulcer [14]. Reactive oxygen species are also involved in the pathogenesis of pylorus ligation ulcer [15]. Pylorus ligation-induced ulcers are due to accumulation of gastric acid and pepsin which lead to autodigestion of gastric mucosa and breakdown of gastric mucosal barrier. The increase in the protein content of the gastric juice in untreated ulcer group indicates the damage to the gastric mucosa as a result of plasma proteins leak into the gastric juice [16]. 

Mucus is secreted by the mucus neck cells and coats the gastric mucosa, thereby preventing physical damage and back diffusion of hydrogen ions [17]. The decreased mucin secretion in control rats indicates the decreased ability of the mucosal membrane to protect the mucosa from physical damage and back diffusion of hydrogen ions. Depletion of the gastric wall mucus has been significantly (*P* < .001) prevented by chloroform fraction. The total acidity and total acid output significantly (*P* < .001) decreased when compared with control group. This data indicates that the antisecretory property involved with the antiulcer activity [18]. Reduction in ulcer index, total acidity, protein, and pepsin content of the gastric fluid and increase in the mucin content indicate the cytoprotective effect of *R. cordifolia.*

Lipid peroxidation is a free radical-mediated process, which has been implicated in a variety of disease states. It involves the formation and propagation of lipid radicals, the uptake of oxygen, and rearrangement of double bonds in unsaturated lipids which eventually results in destruction of membrane lipids. Therefore, it is not surprising that membrane lipids are susceptible to peroxidative attack. In the stomach tissue of rats of aspirin plus pylorus-ligated control group, the MDA level, a lipid peroxidation product, increased significantly compared to the normal group. CF group decreases the MDA level in gastric tissue significantly (*P* < .001) than standard (ranitidine) group. Preventive antioxidants, such as superoxide dismutase (SOD) and catalase (CAT) enzymes, are the first line defence against reactive oxygen species. Reduced glutathione is a major low molecular weight scavenger of free radicals in the cytoplasm and an important inhibitor of free radical-mediated lipid peroxidation [15]. Administration of ME at higher dose (400 mg/kg) and CF showed significant increase in SOD, catalase and reduced glutathione levels as compared to the control animals, which suggest the efficacy in preventing free radical-induced damage.

The above results demonstrated that chloroform fraction was more potent than parent methanol extract at lower dose. As per the phytochemical evidences revealed that the chloroform fraction mainly contains triterpenoids, maybe this activity was because of triterpenoids. As triterpenoids are reported as a good antiulcer and antioxidant compound. 

It could be concluded that *Rubia cordifolia* has both gastroproctective and ulcer-healing properties probably by the two mechanisms: (1) by increasing defensive gastric mucosa and prostaglandin synthesis: (2) Due to antioxidant potential of drug.

## Figures and Tables

**Table 1:** Effect of *R. cordifolia *on gastric acid secretion and ulcer index.

Sr. No.	1	2	3	4	5	6	7	8	9	10	11	12
Group	Normal	Control	Standard	ME 100	ME 200	ME 400	CF 50	CF 100	CF 200	RF 50	RF 100	RF 200
Ulcer Index (%protection)	—	80.63 ± 3.88	21.88 ± 2.01*** (72.86)	68.82 ± 2.22^NS^ (14.64)	66.24 ± 4.48^NS^ (17.84)	54.42 ± 3.54** (32.50)	52.39 ± 1.51** (35.02)	40.82 ± 3.87*** (49.37)	30.87 ± 2.24*** (61.71)	72.89 ± 3.18^NS^ (9.59)	70.82 ± 2.26^NS^ (12.16)	69.65 ± 2.15^NS^ (13.61)
Pepsin (nmols of tyrosine/mL)	—	21.63 ± 1.21	4.62 ± 1.03***	15.85 ± 1.62^NS^	13.32 ± 1.41**	11.82 ± 1.02***	13.82 ± 1.39**	11.28 ± 1.22***	10.62 ± 1.36***	17.27 ± 0.71^NS^	15.62 ± 1.66^NS^	14.27 ± 2.19*
Protein (*μ*g/mL)	—	174.47 ± 9.03	99.42 ± 2.61***	148.26 ± 5.38*	131.27 ± 1.63***	129.53 ± 2.82***	130.62 ± 2.11***	127.26 ± 1.51***	114.42 ± 3.31***	174.56 ± 6.61^NS^	166.36 ± 10.95^NS^	149.26 ± 2.55^NS^
Mucin Content (*μ*g of alcian blue/g wet tissue)	110.2 ± 1.31	61.28 ± 2.22^a^	64.49 ± 1.61^NS^	71.27 ± 1.41^NS^	75.48 ± 2.15**	77.27 ± 3.16**	74.83 ± 2.41*	81.67 ± 2.97***	86.26 ± 3.73***	58.28 ± 2.63^NS^	68.84 ± 2.01^NS^	75.28 ± 2.28*
Total Acidity (meq/L/4 hr)	1088.28 ± 22.29	1939.21 ± 42.71^b^	1195.26 ± 37.03	1844.82 ± 28.77^NS^	1775.82 ± 29.26*	1533.62 ± 15.72**	1545.82 ± 40.83**	1340.32 ± 41.94**	1226.42 ± 38.14**	2070.42 ± 22.49^NS^	1787.42 ± 73.38^NS^	1648.82 ± 29.64**
Total acid Output (meq/L/4 hr)	2459.08 ± 81.97	9318.16 ± 328.23^b^	3068.32 ± 103.24	8398.11 ± 428.97^NS^	7280.47 ± 708.72**	6180.48 ± 172.84**	7574.04 ± 539.46*	5671.92 ± 308.91**	4741.44 ± 41.17**	7871.67 ± 451.58^NS^	7835.26 ± 414.12^NS^	7612.26 ± 359.52*

*N* = 6 normal group= vehicle Na CMC 1% (10 ml/kg, p.o.).

Values are expressed as mean ± SEM. Control group was compared with normal group. Test and standard groups were compared with control group. Statistical comparison was performed using analysis of variance (ANOVA) followed by Turkey's test. ^a^ *P* < .001; ^b^ *P* < .01, when compared with normal group. *P < .05; **P < .01; ***P < .001; NS: nonsignificant, when compared with control groups.

ME100, 200, and 400—methanolic extract of *Rubia cordifolia *100, 200, and 400 mg/kg, p.o,. respectively, CF50,100, and 200—chloroform fraction of *Rubia cordifolia *50, 100, and 200 mg/kg, p.o., respectively, RF50,100, and 200—residual fraction of *Rubia cordifolia *50, 100, and 200 mg/kg, p.o., respectively.

**Table 2:** Effect of *R. cordifolia *on antioxidant parameters in stomach of aspirin + pylorus-ligated rats.

	1	2	3	4	5	6	7	8	9	10	11	12
Group	Normal	Control	Standard	ME 100	ME 200	ME 400	CF 50	CF 100	CF 200	RF 50	RF 100	RF 200
SOD (Units/gm of wet tissue)	204.4 ± 3.61	89.4 ± 2.94^a^	125.2 ± 2.38***	104.48 ± 2.09^NS^	105.24 ± 1.48^NS^	112.36 ± 2.94***	110.84 ± 3.72**	132.92 ± 2.65***	172.06 ± 7.22***	98.54 ± 3.57^NS^	102.68 ± 1.76^NS^	106.14 ± 2.2*
CAT (Units/gm of wet tissue)	45.97 ± 2.12	13.04 ± 1.24^a^	40.35 ± 1.25***	18.96 ± 1.72^NS^	23.73 ± 1.26**	26.3 ± 1.47***	24.3 ± 1.86**	29.73 ± 2.05***	34.4 ± 2.02***	17.18 ± 1.62^NS^	18.84 ± 1.98^NS^	20.35 ± 1.95^NS^
GSH (nmols/gm of wet tissue)	218.4 ± 6.43	94.9 ± 3.42^a^	142.9 ± 6.72***	122.22 ± 4.4*	127.28 ± 4.72**	150.52 ± 3.84***	123.96 ± 4.97**	155.4 ± 4.52***	176.42 ± 4.59***	108.28 ± 4.8^NS^	111.66 ± 5.7^NS^	120.4 ± 4.81*
LPO (nmols of MDA/gm of wet tissue)	139.68 ± 4.27	258.48 ± 3.72^a^	235.64 ± 6.19*	236.8 ± 6.85^NS^	229.24 ± 5.38**	183.46 ± 3.76***	199.66 ± 4.74***	177.76 ± 3.95***	155.94 ± 2.73***	244.94 ± 2.75^NS^	237.76 ± 3.28^NS^	230.1 ± 3.9**

*N* = 6 normal group= vehicle Na CMC 1% (10 ml/ kg, p.o.).

Values are expressed as mean ± SEM. Control group was compared with normal group. Test and standard groups were compared with control group. Statistical comparison was performed using analysis of variance (ANOVA) followed by Turkey's test. ^a^ *P* < .001, when compared with normal group. *P < .05; **P < .01; ***P < .001; NS: nonsignificant, when compared with control groups.

ME100, 200, and 400—methanolic Extract of *Rubia cordifolia *100, 200, and 400 mg/kg, p.o., respectively, CF50,100, and 200—chloroform fraction of *Rubia cordifolia *50, 100, and 200 mg/kg, p.o., respectively, RF50,100, and 200—residual fraction of *Rubia cordifolia *50, 100, and 200 mg/kg, p.o., respectively.
